# Selective Estrogen Receptor Modulators Regulate Dendritic Spine Plasticity in the Hippocampus of Male Rats

**DOI:** 10.1155/2012/309494

**Published:** 2011-11-02

**Authors:** Ignacio González-Burgos, Martha C. Rivera-Cervantes, Dulce A. Velázquez-Zamora, Alfredo Feria-Velasco, Luis Miguel Garcia-Segura

**Affiliations:** ^1^Centro de Investigación Biomédica de Occidente, Guadalajara, Jalisco 44340, Mexico; ^2^CUCBA, Universidad de Guadalajara, Guadalajara, Jalisco 45100, Mexico; ^3^Instituto Cajal, CSIC, 28002 Madrid, Spain

## Abstract

Some selective estrogen receptor modulators, such as raloxifene and tamoxifen, are neuroprotective and reduce brain inflammation in several experimental models of neurodegeneration. In addition, raloxifene and tamoxifen counteract cognitive deficits caused by gonadal hormone deprivation in male rats. In this study, we have explored whether raloxifene and tamoxifen may regulate the number and geometry of dendritic spines in CA1 pyramidal neurons of the rat hippocampus. Young adult male rats were injected with raloxifene (1 mg/kg), tamoxifen (1 mg/kg), or vehicle and killed 24 h after the injection. Animals treated with raloxifene or tamoxifen showed an increased numerical density of dendritic spines in CA1 pyramidal neurons compared to animals treated with vehicle. Raloxifene and tamoxifen had also specific effects in the morphology of spines. These findings suggest that raloxifene and tamoxifen may influence the processing of information by hippocampal pyramidal neurons by affecting the number and shape of dendritic spines.

## 1. Introduction

Selective estrogen receptor modulators (SERMs) either synthetic or natural, such as phytoestrogens, are candidates for the treatment or the prevention of cognitive and affective disorders in men and women [1–5]. Several studies have shown that some synthetic SERMs, such as tamoxifen, raloxifene, or bazedoxifene [6–29], some nonfeminizing estrogens [30–34], and some natural SERMs, such as genistein [35, 36], are neuroprotective in vitro and in vivo. The neuroprotective effects of SERMs are associated with a decrease in the activation of microglia and astroglia and a reduction in brain inflammation [37–43]. In addition, some SERMs have shown to induce neuritic outgrowth in vitro [44, 45], suggesting that these molecules may also affect synaptic connectivity in vivo. Indeed, ERs are involved in the regulation of dendritic spines in the hippocampus of female animals in vivo [46–51], where tamoxifen regulates synaptophysin expression [52]. SERMs are also able to regulate cholinergic, serotonergic, and dopaminergic neurotransmission in female animals [53–56]. However, the effects of SERMs on synaptic connectivity in males have not been adequately explored. Nevertheless, previous studies have shown that SERMs such as raloxifene and tamoxifen are able to counteract hippocampus-dependent cognitive deficits caused by androgen deprivation in male rats [57]. In addition, raloxifene reduces working memory deficits in male rats after traumatic brain injury [20].

To further characterize the mechanisms of action of SERMs in the male brain, we have assessed in this study the effects of tamoxifen and raloxifene on the number and geometry of dendritic spines in CA1 pyramidal neurons of the rat hippocampus.

## 2. Material and Methods

### 2.1. Animals and Treatments

Sprague-Dawley adult male rats were maintained under regular 12 h light-dark cycles (lights on: 07:00–19:00 h) and controlled environmental humidity (45–50%) and temperature (22 ± 2°C). Animals had free access to food and water. All the experimental procedures were conducted to minimize pain or discomfort in the animals and performed in accordance with the NIH guide for Care and Use of Laboratory Animals (NIH Publications no. 80-23, 1996 revised). Protocols were approved by our institutional animal care committee. 

At the age of three months, animals were injected with raloxifene (1 mg/kg; *n* = 6), tamoxifen (1 mg/kg; *n* = 6), or vehicle (20 mg/mL DMSO diluted 3% in saline solution; *n* = 6). Animals were killed 24 h after the injection. 

### 2.2. Golgi Studies

Animals were anesthetized with 30 mg/kg intramuscular ketamine and 50 mg/kg i.p. sodium pentobarbital. Then, animals were perfused intracardially with 100 mL of a washing phosphate-buffered solution (pH 7.4; 0.01 M) containing 1000 IU/L of sodium heparin and 1 g/L of procaine hydrochloride. Then, 200 mL of a fixing phosphate-buffered 4% formaldehyde solution was perfused. Both solutions flowed at a rate of 11.5 mL/min. Each brain remained for at least 48 h in 100 mL of a fresh fixing solution.

**Figure 1:**
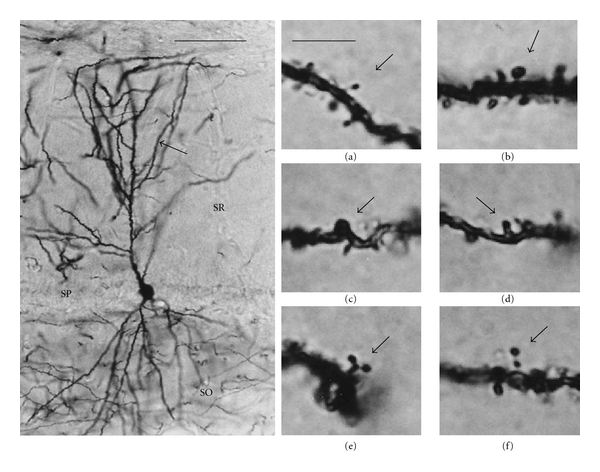
Examples of dendritic spines stained with the Golgi method. Left panel: photomicrograph of a CA1 pyramidal neuron impregnated with a modification of the Golgi method. Spines studied in the present work were counted in a segment 50 *μ*m in length of a secondary dendrite (arrow) protruding from its parent apical dendrite. SO: stratum oriens; SP: stratum pyramidale; SR: stratum radiatum. Scale bar = 100 *μ*m. In the right panels, photomicrographs show representative examples of thin (a), mushroom (b), stubby (c), wide (d), branched (e), and double (f) spines (arrows). Scale bar = 5 *μ*m.

The bilateral dorsal hippocampi were dissected out and impregnated using a modification of the Golgi method [58]. Several coronal slices 100 *μ*m thick were mounted on one slide per animal. Spine numerical density and the proportion of thin, mushroom, stubby, wide, branched, and double spines [59–61] were assessed in CA1 pyramidal neurons. Spines were counted in one 50 *μ*m segment per cell, located in the middle of one of the secondary dendrites that protrude from the apical dendrite (Figure 1). Six CA1 pyramidal neurons were studied per animal. The total number of spines counted was 5,796 in the animals treated with vehicle: 9,295 in the animals treated with raloxifene and 9,180 in the animals treated with tamoxifen.

### 2.3. Statistical Analysis

The one-way ANOVA and Tukey *post hoc* test were used for statistical comparisons of data from spine numerical density. In addition, one-way ANOVA and Bonferroni correction *post hoc* test were used for statistical comparisons of the proportion of the different types of spines. The *n* used for statistical analysis was the number of animals (*n* = 6, per experimental group).

## 3. Results

Raloxifene and tamoxifen increased significantly the total numerical density of dendritic spines compared to control animals (Table 1). Both SERMs increased the numerical density of mushroom, stubby, and wide spines (Table 1). In addition, raloxifene increased the numerical density of thin spines (Table 1). Numerical density of mushroom spines was greater in tamoxifen-treated rats than in raloxifene-treated animals. In contrast, thin and wide spines were less numerous in the tamoxifen group than in raloxifene-treated animals (Table 1).

The experimental treatments also resulted in changes in the proportion of different spine morphologies. The proportion of thin spines was reduced in the animals treated with raloxifene. Furthermore, raloxifene increased the proportion of stubby and wide spines and did not significantly affect the proportion of mushroom, branched and double spines (Table 2).

As observed for raloxifene, the proportion of thin spines was also reduced in the animals treated with tamoxifen. In contrast, mushroom and stubby spines were seen in greater proportion in animals treated with tamoxifen than in control animals. Tamoxifen had no significant effects in the proportion of wide, branched, and double spines (Table 2). The animals treated with tamoxifen showed a higher proportion of mushroom spines than those treated with raloxifene (Table 2).

## 4. Discussion

Our present findings indicate that some SERMs, such as raloxifene and tamoxifen, affect the number of dendritic spines in male rats. This action of SERMs may affect the processing of novel information used in memory formation [62].

In addition to increase the numerical density of spines, raloxifene and tamoxifen also affected spine geometry. Both SERMs increased the numerical density of stubby, mushroom, and wide spines. In addition, raloxifene increased the number of thin spines. However, both SERMs reduced the proportion of thin dendritic spines. Dendritic spine morphology affects the diffusion and compartmentalization of membrane-associated proteins [63] and the expression of AMPA receptors [64–67]. In particular, the length of the spine neck seems to be a key regulator of spinodendritic Ca2+ signaling [68–72] and of the transmission of membrane potentials [73]. In consequence, the geometry of dendritic spines may influence the processing of synaptic impulses [74–79]. Our findings suggest, therefore, that raloxifene and tamoxifen, decreasing the proportion of thin dendritic spines, may influence the processing of information by hippocampal pyramidal neurons. In addition, the action of raloxifene and tamoxifen presents some differences that may have functional significance. Tamoxifen, but not raloxifene, increased the proportion of mushroom spines. Thus, the animals treated with tamoxifen had an increased numerical density and proportion of mushroom spines compared to animals treated with raloxifene. Mushroom spines may be involved in the management of previously acquired information since they have larger postsynaptic densities [80] and express higher levels of AMPA receptors [64–67]. Therefore, the synapses on mushroom spines are functionally stronger [78] and it has been suggested that these spines would sustain memory storage [78, 81, 82]. 

The induction of plastic changes in dendritic spines by raloxifene and tamoxifen may be linked with the precognitive effects of these molecules in male rats [20, 57]. However, the possible impact of raloxifene and tamoxifen on cognitive decline in men remains to be adequately explored, in particular in association with neurodegenerative diseases. For instance, both SERMs increase the levels of luteinizing hormone (LH) in men [83] and it has been proposed that elevated levels of LH may contribute to Alzheimer's disease pathogenesis [84]. Indeed, leuprolide acetate, a GnRH agonist that lower serum levels of LH, has been shown to improve cognitive performance and decrease amyloid-*β* deposition in a mouse transgenic model of Alzheimer's disease [85].

## 5. Conclusions

The findings of this study indicate that raloxifene and tamoxifen, two SERMs currently used in clinical treatments, promote an increase in the numerical density of dendritic spines and changes in spine geometry in the hippocampus of male rats. These findings, together with the regulation exerted by tamoxifen and raloxifene on hippocampus-dependent cognitive function in male rats [57], suggest that SERMs may influence the processing of information by male hippocampal pyramidal neurons by affecting the number and shape of dendritic spines.

## Figures and Tables

**Table 1:** Numerical density of dendritic spines in hippocampal CA1 pyramidal neurons of male rats 24 hours after the treatment with vehicle, raloxifene, or tamoxifen.

	Vehicle	Raloxifene	Tamoxifen
Total spines	161.0 ± 5.0	258.2 ± 3.0^a^	255.0 ± 4.0^b^
Thin	74.8 ± 2.8	89.6 ± 3.8^a^	78.0 ± 3.6^c^
Mushroom	50.4 ± 1.8	84.6 ± 3.2^a^	92.8 ± 1.6^bc^
Stubby	28.6 ± 1.4	65.8 ± 1.8^a^	71.0 ± 2.2^b^
Wide	6.2 ± 0.6	15.6 ± 1.0^a^	11.8 ± 1.2^bc^
Branched	0.4 ± 0.1	0.6 ± 0.2	0.6 ± 0.1
Double	0.1 ± 0.04	0.04 ± 0.04	0.4 ± 0.1

Data represent mean ± SEM of the number of dendritic spines per 100 *μ*m dendritic segment from 6 animals in each experimental group. ^a–c^Significant differences, *P* < 0.05; ^a^raloxifene versus Vehicle;  ^b^tamoxifen versus. Vehicle; ^c^tamoxifen versus Raloxifene.

**Table 2:** Proportion (%) of the different types of dendritic spines in hippocampal CA1 pyramidal neurons 24 hours after the treatment with vehicle, raloxifene, or tamoxifen.

	Vehicle	Raloxifene	Tamoxifen
Thin	46.4	34.7^a^	30.5^b^
Mushroom	31.3	32.7	36.3^bc^
Stubby	17.7	25.4^a^	27.8^b^
Wide	3.8	6.0^a^	4.6
Branched	0.2	0.2	0.2
Double	0.07	0.01	0.1

Data represent means from 6 animals in each experimental group. ^a–c^Significant differences, *P* < 0.05; ^a^raloxifene versus Vehicle;  ^b^tamoxifen versus Vehicle;  ^c^tamoxifen versus raloxifene.
